# Association of colorectal polyps and cancer with low-dose persistent organic pollutants: A case-control study

**DOI:** 10.1371/journal.pone.0208546

**Published:** 2018-12-06

**Authors:** Yu-Mi Lee, Se-A Kim, Gyu-Seog Choi, Soo-Yeun Park, Seong Woo Jeon, Hyun Seok Lee, Su-Jin Lee, Somi Heo, Duk-Hee Lee

**Affiliations:** 1 Department of Preventive Medicine, School of Medicine, Kyungpook National University, Daegu, Republic of Korea; 2 Department of Biomedical Science, Graduate School, Kyungpook National University, Daegu, Republic of Korea; 3 BK21 Plus KNU Biomedical Convergence Program, Department of Biomedical Science, Kyungpook National University, Daegu, Republic of Korea; 4 Department of Surgery, School of Medicine, Kyungpook National University, Daegu, Republic of Korea; 5 Department of Internal Medicine, School of Medicine, Kyungpook National University, Daegu, Republic of Korea; 6 Graduate School of Department of Public Health, Kyungpook National University, Daegu, Republic of Korea; University of Kwazulu-Natal, SOUTH AFRICA

## Abstract

**Background:**

Low-dose persistent organic pollutants (POPs) have recently been linked to immunosenescence, a key mechanism in carcinogenesis, as well as many aging-related chronic diseases. Since feces are the main excretion route of POPs, the large intestine is a potential target organ for these pollutants. We performed a case-control study to evaluate whether exposure to low-dose POPs is related to the risk of colorectal polyps and cancer.

**Methods:**

A total of 277 participants were recruited from one hospital: 99 cancer patients, 102 polyp patients, and 76 control subjects. As typical examples of POPs, we measured the serum concentrations of organochlorine pesticides (OCPs) and polychlorinated biphenyls (PCBs).

**Results:**

Across the tertiles of the summary measure of POPs, the adjusted odds ratios (ORs) of colorectal polyps and cancer were 2.8 (1.2–6.8) (*P*_trend_ = 0.01) and 3.0 (1.0–8.8) (*P*_trend_ = 0.02), respectively, for subjects in the highest tertile. When OCPs and PCBs were analyzed separately, OCPs were linked to an increased risk of both polyps and cancer; the adjusted ORs were 2.3 (0.9–5.7) (*P*_trend_ = 0.05) for polyps and 3.6 (1.1–11.8) (*P*_trend_< 0.01) for cancer. However, PCBs were only significantly associated with a high risk of polyps but not cancer; the adjusted OR was 2.8 (1.2–6.6) (*P*_trend_ = 0.01).

**Conclusion:**

Chronic exposure to low-dose POPs may be associated with an increased risk of colorectal polyps and cancer. Our findings suggest the carcinogenic potential of strong lipophilic chemical mixtures such as POPs which are accumulated in adipose tissue, released to circulation, and eliminated through feces.

## Introduction

Persistent organic pollutants (POPs) refer to hundreds of strong lipophilic chemicals with long half-lives, which bioaccumulate in food webs and adipose tissue of living organisms and are resistant to environmental degradation [1]. Typical examples of POPs include organochlorine pesticides (OCPs) and polychlorinated biphenyls (PCBs). Recently, many aging-related chronic diseases, including diabetes and dementia in general populations, have been linked to the chronic exposure to low-dose POPs [1–3]. In addition, low-dose OCPs in a contemporary general population is suggested as a new risk factor for T cell immunosenescence, which is mechanistically linked to the risk of developing cancer [4].

At present, PCBs are classified as definite human carcinogens based on epidemiological evidence linking PCBs and melanoma [5]. However, human evidence on other POPs is inconsistent or sparse. For example, OCPs are considered “probably” or “possibly” carcinogenic to humans because of inconsistent results obtained in human studies [6]. In addition, there is still criticism on the classification of PCBs as human carcinogens because more consistent results were obtained in general populations exposed to low background doses of PCBs than in those performed among occupationally exposed workers or among people accidentally exposed to high levels of PCBs [7].

Traditionally, researchers have assumed that it is best to evaluate the carcinogenic potential of chemicals among populations with high-dose exposure to these chemicals. However, it has been recently reported that many chemicals involved in carcinogenesis may not show linearity [8]. Under non-linearity, general populations with low-dose exposure would be more suitable than those with high-dose exposure because the presence of an unexposed group (or study subjects is as close as possible to an unexposed group) is critical to validly evaluate the risk in humans [9]. Therefore, the role of POPs as human carcinogens needs to be reevaluated in contemporary general populations with low-dose exposure to these pollutants.

Among various cancer sites, the large intestine is a likely target organ because its mucosa is continuously exposed to low-dose POPs, which are mainly eliminated from the body in feces, through bile and passive transluminal diffusion [10, 11]. Until now, human studies conducted among occupational workers who were heavily exposed to high doses of OCPs or PCBs have reported very inconsistent results, from inverse to positive associations with colorectal cancer, when compared with the risks reported for general populations [12–18]. Furthermore, two case-control studies, which were performed in general populations with relatively high-dose exposure, have reported weak or no associations [19, 20]. However, associations between POPs and colorectal cancer need to be reevaluated in a general population with low-dose exposure for the reasons we discussed above.

Moreover, it is worth evaluating whether POPs are related to the risk of colorectal polyps because most colorectal cancers are preceded by colorectal polyps [21, 22]. At present, known risk factors of colorectal polyps are mostly related to lifestyle, such as cigarette smoking and obesity, although the results differ depending on the histological type of polyps [23]. To the best of our knowledge, there have been no studies evaluating the association between POPs and colorectal polyps.

Therefore, this hospital-based case-control study was performed to evaluate the associations of serum concentrations of OCPs and PCBs with the risks of colorectal polyps and cancer among a general population with low-dose exposure.

## Materials and methods

### Study participants

Study participants were recruited at the outpatient clinic and routine health checkup center of the Kyungpook National University Chilgok Hospital, Daegu, Korea, from April 2014 to May 2016. Patients with newly diagnosed colorectal cancer were enrolled in the colorectal cancer group. Among people who underwent colonoscopy for the purpose of a health checkup, patients diagnosed with colorectal polyps were enrolled in the colorectal polyp group, and those with a normal status were enrolled in the control group.

Participants who met at least one of the following criteria were excluded: 1) diagnosed with inflammatory bowel disease; and 2) diagnosed with familial adenomatous polyposis. In the case of the control group, we further excluded subjects with any history of polypectomy. Assuming an α-error of 0.05, a power of 80%, a 1:1 ratio of case to control, a 20–60% exposure rate among controls, and an odds ratio of 2.5, the derived sample size range was 76–94 per group. The final sample sizes were 99 in the cancer group, 102 in the polyp group, and 76 in the control group. Written informed consent was obtained from each participant. The Institutional Review Board of the Kyungpook National University Hospital reviewed and approved the study protocol (IRB no. KNUH 2014-11-037). The study was reported according to the STROBE guidelines (S1 Table).

### General and clinical characteristics

Information on the demographic characteristics, history of diseases, family history of diseases, and health-related behaviors was surveyed by a trained interviewer using a structured questionnaire. The height was measured in a standing position without shoes, and the weight was measured with light clothing. The body mass index (BMI, kg/m^2^) was calculated by dividing the weight in kilograms by the height in meters squared. The extent of cigarette smoking was measured as a combination of the number of cigarette packs smoked per day and the number of years of smoking (pack-years). Physical activity was categorized as low, moderate, and high using a short, self-administered Korean Short Form questionnaire from the International Physical Activity Questionnaire [24] scoring protocol. Dietary behaviors were surveyed for 112 food items using the semi-quantitative food frequency questionnaire (FFQ) used in the Korea National Health and Nutrition Examination Survey (KNHANES), developed by the Korea Centers for Disease Control and Prevention [25]. Based on the frequency and amount of food intake, which were determined using the FFQ, the amounts of alcohol consumption (g/day), red meat consumption (g/day), fiber intake (g/day) were estimated.

Venous blood samples were collected after overnight fasting for at least 8 h. Approximately 10 mL of blood was drawn from each participant. The samples were stored in a freezer at −70 °C until analysis. Total cholesterol and triglyceride levels were measured by an enzymatic colorimetric assay using a Cobas 8000 C702 chemistry autoanalyzer (Roche, Germany).

### Concentrations of POPs in serum

Serum concentrations of OCPs and PCBs were quantified by high-resolution gas chromatography with high-resolution mass spectrometry (AutoSpec Premier; Waters, Milford, MA, USA) at a laboratory of Hanyang University (Ansan, Korea). The limit of detection (LOD) was defined as the concentration that produced a signal equivalent to three times the baseline noise. The concentrations of POPs that were below LOD were substituted with a constant value equal to one-third of LOD.

Of 17 OCPs and 19 PCBs tested, the statistical analysis included 11 OCPs and 14 PCBs whose concentrations were above LOD in at least 10% of the participants. The following 11 OCPs were included: β-hexachlorocyclohexane, *o*,*p′*-dichlorodiphenyldichloroethylene (DDE), *p*,*p′*-DDE, *o*,*p′*-dichlorodiphenyltrichloroethane (DDT), *p*,*p′*-DDT, *trans*-chlordane, oxychlordane, *trans*-nonachlor, *cis*-nonachlor, heptachlor epoxide, and heptachlor. The 14 PCBs included were PCB 18, PCB 28, PCB 33, PCB 52, PCB 101, PCB 105, PCB 118, PCB 138, PCB 153, PCB 170, PCB 180, PCB 187, PCB 194, and PCB 199. The detection rates and distribution of POP concentrations among the participants are presented in S2 Table. Lipid-standardized concentrations of POPs, which were primarily used in this study, were calculated by dividing wet-weight concentrations of POPs by total lipids (mg/dL): total lipids = 2.27 × (total cholesterol + triglycerides + 62.3) [26]. The results on wet concentrations are also presented in the Results section.

### Statistical analysis

First, we compared serum concentrations of all individual OCP and PCB compounds among the three groups using general linear models. Then, we estimated odds ratios (ORs) of an increased risk of colorectal polyps or cancer across tertiles of serum concentrations of OCPs or PCBs using polychotomous logistic regression. For these analyses, we focused on summary measures of POPs rather than on individual compounds because general populations are simultaneously exposed to a mixture of all these chemicals, and serum concentrations of individual compounds are positively correlated with the level of exposure, especially for compounds belonging to the same subclass. It is important to note that interpretations based on individual POP compounds, which have been performed in most epidemiological studies about POPs, may be misleading [9].

To estimate the summary measures of POPs, we added rank orders of individual compounds belonging to each subclass (S1 Fig). Summary measures of OCPs (∑OCPs) and PCB (∑PCBs) were calculated by summing up the ranks of the 11 individual OCPs and 14 individual PCBs, respectively. Further, we calculated several additional summary measures, including those for DDT congeners (∑DDTs = *o*,*p′*-DDE + *p*,*p′*-DDE + *o*,*p′*-DDT + *p*,*p′*-DDT); chlordane congeners (∑chlordanes = *trans*-chlordane + oxychlordane + *trans*-nonachlor + *cis*-nonachlor); heptachlor congeners (∑heptachlor = heptachlor epoxide + heptachlor). PCBs were categorized by the degree of chlorination, which it is closely related to persistence; more chlorinated PCBs have longer half-lives than less chlorinated PCBs [27]. PCB congeners with three or four chlorides (∑low-chlorinated PCBs = PCB 18 + PCB 28 + PCB 33 + PCB 52); PCB congeners with five or six chlorides (∑mid-chlorinated PCBs = PCB 101 + PCB 105 + PCB 118 + PCB 138 + PCB 153); and PCB congeners with seven or more chlorides (∑high-chlorinated PCBs = PCB 170 + PCB 180 + PCB 187 + PCB 194 + PCB 199). In the case of hexachlorocyclohexanes, only β-hexachlorocyclohexane was used because there were no other congeners with a detection rate of 10% or higher.

Although absolute concentration-based summary measures seem to be intuitively more sensible, they are often determined by one or two individual compounds with very high absolute concentrations and, therefore, are somewhat meaningless as summary measures [1]. On the other hand, rank-based summary measures enable equal contributions from all constituents. This approach is especially important when compounds with low detection rates and low absolute concentrations are more significant than compounds with high detection rates and high absolute concentrations, which can be expected under the non-linear dose-response relationship. The advantage of rank-based summary measures of POPs over other methods has been discussed in detail elsewhere [1].

Known risk factors of colorectal polyps or cancer were considered covariates. They were the age, sex, family history of colorectal cancer (yes/no), BMI, cigarette smoking (pack-years), alcohol drinking (g/day), physical activity (low/moderate/high), red meat consumption (g/day), fiber intake (g/day) and physician-diagnosed diabetes (yes/no). In addition, we considered the weight change during the past year as a possible confounder because weight loss is one of the common symptoms among cancer patients [28], and it increases serum concentrations of POPs because of their release from adipose tissue [29].

When wet concentrations of POPs were used, serum cholesterol and triglycerides were considered covariates. All analyses were carried out using the SAS 9.4 software (SAS Institute, Cary, NC, USA). *P*-values of less than 0.05 were considered statistically significant.

## Results

### General characteristics

General characteristics of the study participants are shown in Table 1. The mean ages of the controls, polyp cases, and cancer cases were 52.1, 55.6, and 65.9 years, respectively. Males constituted 46.1%, 70.6%, and 55.6% of the controls, polyp cases, and cancer cases, respectively. BMI of the polyp cases was significantly higher than that of the controls and cancer cases, based on the post-hoc test. Cigarette smoking and alcohol consumption were significantly higher among the polyp cases than among the controls (post-hoc test). The prevalence of hypertension and diabetes tended to be higher in the polyp and cancer groups than in the control group. The physical activity, red meat consumption, fiber intake, and family history of colorectal cancer were not significantly different among the groups. The cancer cases were diagnosed with stage I (11.1%), stage II (36.4%), stage III (49.5%), and stage IV (3.0%) disease. The histologic types of polyps in the polyp cases were adenoma (62.7%), hyperplastic polyps (8.8%), and serrated adenoma (4.9%). The correlation coefficients between summary measures of OCPs and PCBs are presented in S3 Table.

**Table 1:** General characteristics of subjects from the control, colorectal polyp, and colorectal cancer groups.

Characteristics		Control group (n = 76)	Colorectal polyp group (n = 102)	Colorectal cancer group (n = 99)	*P*-value*
		Mean ± standard deviation			
Age (years)		52.1 ± 7.8 (range: 40–70)	55.6 ± 8.9^a^ (range: 40–75)	65.9 ± 10.0^a^^,^^b^ (range: 34–84)	<0.01
Body mass index (kg/m^2^)		23.6 ± 2.7	24.7 ± 3.0^a^	23.4 ± 2.9^b^	<0.01
Cigarette smoking (pack-year)		9.7 ± 19.8	20.4 ± 23.5^a^	18.0 ± 25.8	<0.01
Alcohol consumption (g/day)		9.2 ± 21.0	25.8 ± 34.6^a^	16.5 ± 29.7	<0.01
Red meat consumption (g/day)		60.2 ± 81.0	90.4 ± 129.2	61.1 ± 91.1	0.08
Fiber intake (g/day)		31.8 ± 16.2	35.6 ± 16.5	30.7 ± 17.6	0.10
Fasting blood glucose (mg/dL)		92.7 ± 14.3	100.2 ± 30.2	103.4 ± 28.5^b^	0.03
Total cholesterol (mg/dL)		194.0 ± 36.9	191.4 ± 42.8	159.5 ± 38.1^a^^,^^b^	<0.01
Triglycerides (mg/dL)		110.7 ± 58.8	158.1 ± 209.0	114.5 ± 63.6	0.03
		n (%)			
Males		35 (46.1)	72 (70.6)	55 (55.6)	<0.01
Physical activity	low	29 (38.2)	34 (33.3)	12(12.1)	<0.01
	moderate	27 (35.5)	43 (42.2)	58(58.6)	
	high	20 (26.3)	25 (24.5)	29(29.3)	
Physician-diagnosed hypertension		11 (14.5)	33 (32.4)	46 (46.5)	<0.01
Physician-diagnosed diabetes		5 (6.6)	16 (15.7)	25 (25.3)	<0.01
Family history of colorectal cancer		6 (7.9)	14 (13.7)	10 (10.1)	0.45
Cancer stage	I	–	–	11 (11.1)	
	II	–	–	36 (36.4)	
	III	–	–	49 (49.5)	
	IV	–	–	3 (3.0)	
Histologic type of polyps^c^	Adenoma	–	64 (62.7)	–	
	Serrated adenoma	–	5 (4.9)	–	
	Hyperplastic polyps	–	9 (8.8)	–	
	Adenoma + serrated adenoma	–	3 (2.9)	–	
	Adenoma + hyperplastic polyp	–	16 (15.7)	–	
	Others	–	5 (4.9)	–	

*A chi-squared test was used for categorical variables, and one-way analysis of variance was used for continuous variables.

^a^*P* < 0.05 compared with controls;

^b^*P* < 0.05 compared with colorectal polyp cases (Scheffe’s post-hoc test).

^c^Three polyps of those removed during polypectomy were examined for each study participant.

### Comparison of serum concentrations of POPs among three groups

In Table 2, we compared serum concentrations of the 11 OCP compounds and 14 PCB compounds. Among the 11 OCPs, five compounds (*o*,*p′*-DDE, oxychlordane, *cis*-nonachlor, heptachlor epoxide, and heptachlor) showed significant differences among the groups, and one compound (β-hexachlorocyclohexane) showed marginally significant differences. OCPs with the highest concentrations in the cancer group were *o*,*p′*-DDE, oxychlordane, heptachlor epoxide, and *cis*-nonachlor, while those with the highest concentrations in the polyp group were heptachlor and β-hexachlorocyclohexane. The detection rates of these OCPs, except β-hexachlorocyclohexane, were generally low (in the range of 13.4–70.4%).

**Table 2:** Comparison of adjusted geometric means of lipid-adjusted concentrations of organochlorine pesticides and polychlorinated biphenyls among the control, colorectal polyp, and colorectal cancer groups.

POPs	Detection rate (%)	Geometric mean* ± standard error (ng/g of lipid)			*P*-value
		Controls (n = 76)	Colorectal polyp cases (n = 102)	Colorectal cancer cases (n = 99)	
OCPs					
β-hexachlorocyclohexane	96.8	12679.1 ± 1562.3	18610.2 ± 1900.7	16696.9 ± 1873.3	0.05
*o*,*p'*-DDE	13.4	189.8 ± 22.6	234.1 ± 23.1	320.0 ± 34.6 ^b^	0.01
*p*,*p'*-DDE	99.6	174964.9 ± 17319.1	184837.3± 15165.3	176523.1 ± 15909.4	0.89
*o*,*p'*-DDT	13.7	202.2 ± 22.4	246.7 ± 22.6	269.1 ± 27.1	0.19
*p*,*p'*-DDT	91.3	7155.1 ± 1122.4	6662.8 ± 866.3	7546.8 ± 1077.9	0.82
*trans*-chlordane	12.6	90.9 ± 10.6	105.6 ± 10.2	100.1 ± 10.6	0.61
oxychlordane	70.4	750.9 ± 163.2	523.4 ± 94.3	4079.3 ± 807.3 ^b^^,^^c^	<0.01
*trans*-nonachlor	96.4	5902.4±747.2	6704.3±703.4	7612.3±877.4	0.39
*cis*-nonachlor	58.8	556.3±118.5	335.9±59.3	738.2±143.1 ^c^	0.01
heptachlor epoxide	57.4	609.4 ± 112.7	719.6 ± 110.3	2179.2 ± 366.9 ^b^^,^^c^	<0.01
heptachlor	17.0	188.7 ± 30.2	448.4 ± 59.5 ^a^	260.6 ± 38.0 ^c^	<0.01
PCBs					
PCB18	36.1	121.8 ± 15.3	193.8 ± 20.2 ^a^	217.9 ± 24.9 ^b^	<0.01
PCB28	21.7	113.5 ± 17.7	292.2 ± 37.9 ^a^	109.1 ± 15.5 ^c^	<0.01
PCB33	12.6	81.4 ± 8.9	187.6 ± 17.0 ^a^	91.3 ± 9.1 ^c^	<0.01
PCB52	30.0	132.9 ± 19.8	211.4 ± 26.1 ^a^	165.3 ± 22.4	0.05
PCB101	24.5	123.6 ± 14.3	156.7 ± 15.1	129.0 ± 13.6	0.21
PCB105	68.6	569.4 ± 92.0	562.7 ± 75.4	487.1 ± 71.7	0.75
PCB118	95.7	3466.4 ± 446.6	3621.6 ± 386.7	3404.8 ± 399.4	0.92
PCB138	92.4	13939.8 ± 2111.9	9868.4 ± 1239.2	7129.2 ± 983.4 ^b^	0.01
PCB153	100.0	21956.7 ± 1461.8	23455.9 ± 1294.4	19387.7 ± 1175.2	0.09
PCB170	89.9	3076.5 ± 439.5	3038.3 ± 359.8	4236.2 ± 551.1	0.16
PCB180	99.6	11150.3 ± 968.0	14962.5 ± 1076.7 ^a^	12429.6 ± 982.5	0.03
PCB187	95.3	5202.8 ± 595.6	5736.4 ± 544.3	5289.9 ± 551.4	0.76
PCB194	55.6	918.6 ± 169.8	724.7 ± 111.1	937.9 ± 157.9	0.46
PCB199	71.1	964.7 ± 139.5	1464.6 ± 175.5	1252.2 ± 164.9	0.08

*Adjusted for age, sex, family history, body mass index, cigarette smoking, alcohol drinking, physical activity, meat consumption, diabetes and fiber intake.

^a^*P* < 0.05 controls vs. colorectal polyp group;

^b^*P* < 0.05 controls vs. colorectal cancer group;

^c^*P* < 0.05 colorectal polyp group vs. colorectal cancer group (Scheffe’s post-hoc test).

DDE, dichlorodiphenyldichloroethylene; DDT, dichlorodiphenyltrichloroethane; OCP, organochlorine pesticide; PCB, polychlorinated biphenyl; POP, persistent organic pollutant.

Compared with OCPs, PCBs tended to show the highest concentrations in the polyp group but not in the cancer group (Table 2). Specifically, low-chlorinated PCBs with low detection rates, such as PCB 18, PCB 28, PCB 33, and PCB 52, showed significantly higher concentrations in the polyp group than those in the control group. Among high-chlorinated PCBs, only PCB 180 showed a higher concentration in the polyp group than that in the control group. On the other hand, PCB 138 showed a higher concentration in the control group than those in the polyp and cancer groups.

**Fig 1:**
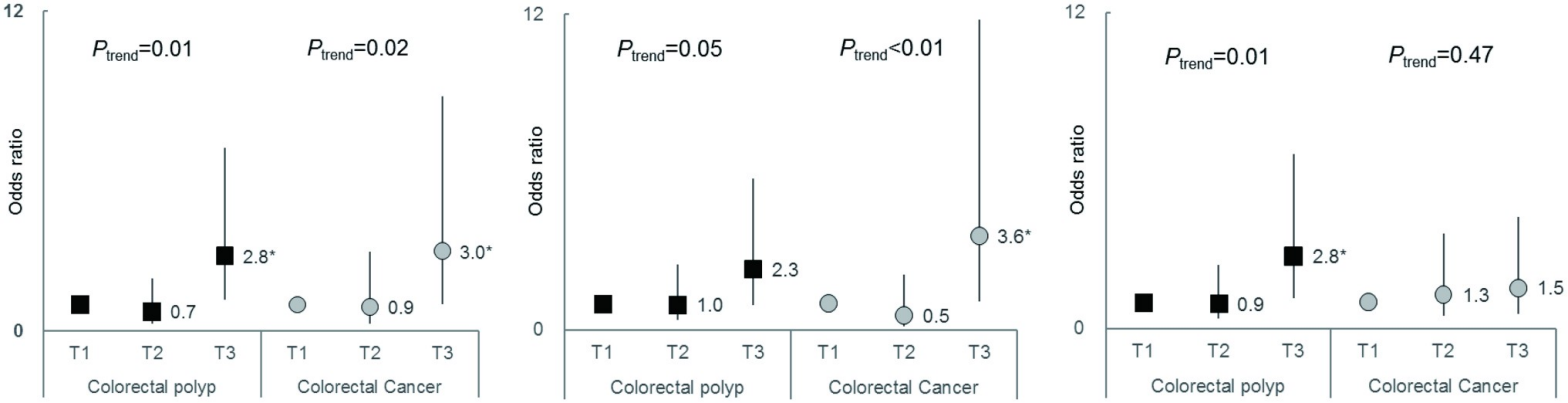
Associations between the summary measures of persistent organic pollutants, organochlorine pesticides, and polychlorinated biphenyls and the risk of colorectal polyps or cancer, calculated using polychotomous logistic regression. (A) ∑POPs. (B) ∑OCPs. (C) ∑PCBs Odds ratios adjusted for age, sex, family history, body mass index, cigarette smoking, alcohol drinking, physical activity, meat consumption, diabetes, and fiber intake. *Confidence interval does not contain 1. ∑POPs = ∑OCPs + ∑PCBs; ∑OCPs = β-hexachlorocyclohexane + ∑DDTs + ∑chlordanes + ∑heptachlor; ∑PCBs = ∑low-chlorinated PCBs + ∑mid-chlorinated PCBs + ∑high-chlorinated PCBs. DDT, dichlorodiphenyltrichloroethane; OCP, organochlorine pesticide; PCB, polychlorinated biphenyl; POP, persistent organic pollutant.

Next, we evaluated the risk of colorectal polyps or cancer across tertiles of summary measures of POPs, OCPs, and PCBs using polychotomous logistic regression. When the data were adjusted for known risk factors of colorectal polyps or cancer, ORs of colorectal polyps and cancer for the subjects in the highest tertile of the summary measure of POPs were 2.8 (1.2–6.8) (*P*_trend_ = 0.01) and 3.0 (1.0–8.8) (*P*_trend_ = 0.02), respectively (Table 3, Model 3, Fig 1). When further adjustment was made for the weight changes during the past year, the results did not change.

**Table 3:** Associations between the summary measures of persistent organic pollutants, organochlorine pesticides, and polychlorinated biphenyls and the risk of colorectal polyps or cancer, calculated using polychotomous logistic regression (Odds ratios and 95% confidence intervals).

Measures	Model	Dependent variables: colorectal polyps			*P*_trend_	Dependent variables: colorectal cancer			*P*_trend_
		1^st^ tertile	2^nd^ tertile	3^rd^ tertile		1^st^ tertile	2^nd^ tertile	3^rd^ tertile	
∑POPs	cases/controls	18/26	16/26	68/24		9/26	25/26	65/24	
	Model 1	1.0	0.6 (0.3–1.6)	2.8 (1.2–6.2)	<0.01	1.0	1.0 (0.3–3.2)	3.3 (1.2–9.5)	0.01
	Model 2	1.0	0.7 (0.3–1.9)	2.7 (1.1–6.3)	0.01	1.0	0.9 (0.3–3.0)	2.9 (1.0–8.6)	0.02
	Model 3	1.0	0.7 (0.3–2.0)	2.8 (1.2–6.8)	0.01	1.0	0.9 (0.3–3.0)	3.0 (1.0–8.8)	0.02
∑OCPs	cases/controls	18/26	24/26	60/24		7/26	15/26	77/24	
	Model 1	1.0	1.0 (0.4–2.5)	2.8 (1.2–6.5)	0.01	1.0	0.5 (0.1–1.9)	3.7 (1.2–11.3)	<0.01
	Model 2	1.0	1.0 (0.4–2.5)	2.3 (0.9–5.7)	0.04	1.0	0.5 (0.1–2.0)	3.4 (1.0–11.1)	<0.01
	Model 3	1.0	1.0 (0.4–2.5)	2.3 (0.9–5.7)	0.05	1.0	0.5 (0.1–2.1)	3.6 (1.1–11.8)	<0.01
∑PCBs	cases/controls	17/26	18/26	67/24		18/26	35/26	46/24	
	Model 1	1.0	0.8 (0.3–1.9)	2.8 (1.2–6.4)	<0.01	1.0	1.2 (0.5–3.3)	1.7 (0.6–4.3)	0.38
	Model 2	1.0	0.9 (0.3–2.2)	2.6 (1.1–6.1)	0.01	1.0	1.3 (0.5–3.7)	1.5 (0.6–4.1)	0.50
	Model 3	1.0	0.9 (0.4–2.4)	2.8 (1.2–6.6)	0.01	1.0	1.3 (0.5–3.6)	1.5 (0.6–4.2)	0.47

Model 1, adjusted for age and sex; Model 2, further adjusted for family history, body mass index, cigarette smoking, alcohol drinking, and physical activity; Model 3, further adjusted for meat consumption, diabetes and fiber intake. ∑POPs = ∑OCPs + ∑PCBs; ∑OCPs = β-hexachlorocyclohexane + ∑DDTs + ∑chlordanes + ∑heptachlor; ∑PCBs = ∑low-chlorinated PCBs + ∑mid-chlorinated PCBs + ∑high-chlorinated PCBs. DDT, dichlorodiphenyltrichloroethane; OCP, organochlorine pesticide; PCB, polychlorinated biphenyl; POP, persistent organic pollutant.

When OCPs and PCBs were analyzed separately, OCPs showed a relationship to the increased risk of both colorectal polyps and cancer; the adjusted ORs were 2.3 (0.9–5.7) (*P*_trend_ = 0.05) and 3.6 (1.1–11.8) (*P*_trend_ < 0.01). However, PCBs were significantly associated only with a high risk of polyps but not cancer; the adjusted OR was 2.8 (1.2–6.6) (*P*_trend_ = 0.01).

In analyses focused on subgroups of OCPs (Table 4), the β-hexachlorocyclohexane, DDT, chlordane, and heptachlor subgroups showed similar patterns. Among them, the summary measure of the heptachlor subgroup showed the strongest associations; the adjusted OR for the subjects with serum concentrations in the third tertile was 6.5 (2.7–15.6). Colorectal polyps had the strongest association with β-hexachlorocyclohexane; the adjusted OR for the subjects with serum concentrations in the third tertile was 6.0 (2.1–16.9). When PCBs were classified into low-, middle-, and high-chlorinated PCBs, the summary measure of the low-chlorinated PCBs showed significant correlations with both colorectal polyps and cancer (Table 4). The adjusted ORs of colorectal polyps and cancer were 2.5 (1.1–5.4) (*P*_trend_ = 0.02) and 4.0 (1.6–10.0) (*P*_trend_ < 0.01), respectively, for the subjects in the highest tertile of the summary measure of the low-chlorinated PCBs.

**Table 4:** Associations between the summary measures of subgroups of organochlorine pesticides and polychlorinated biphenyls and the risk of colorectal polyps or cancer, calculated using polychotomous logistic regression (Odds ratios and 95% confidence intervals).

Measures	Model	Dependent variables: colorectal polyps			*P*_trend_	Dependent variables: colorectal cancer			*P*_trend_
		1^st^ tertile	2^nd^ tertile	3^rd^ tertile		1^st^ tertile	2^nd^ tertile	3^rd^ tertile	
OCP subgroups									
β-hexachlorocyclohexane	cases/controls	17/26	27/26	58/24		9/26	27/26	63/24	
	Model 1	1.0	2.1 (0.9–5.1)	4.7 (1.9–11.7)	<0.01	1.0	4.1 (1.3–12.8)	2.6 (0.8–7.8)	0.24
	Model 2	1.0	2.1 (0.8–5.3)	5.5 (2.0–15.1)	<0.01	1.0	5.1 (1.5–17.0)	3.7 (1.1–12.5)	0.11
	Model 3	1.0	2.2 (0.8–5.8)	6.0 (2.1–16.9)	<0.01	1.0	5.3 (1.5–17.9)	3.7 (1.1–12.5)	0.11
∑DDTs	cases/controls	28/26	21/26	53/24		11/26	26/26	62/24	
	Model 1	1.0	0.6 (0.2–1.3)	1.4 (0.6–3.0)	0.28	1.0	1.1 (0.4–3.2)	2.8 (1.0–7.5)	0.03
	Model 2	1.0	0.6 (0.2–1.4)	1.2 (0.5–2.7)	0.59	1.0	1.2 (0.4–3.6)	2.5 (0.9–7.0)	0.07
	Model 3	1.0	0.6 (0.2–1.5)	1.2 (0.5–2.9)	0.52	1.0	1.1 (0.4–3.5)	2.4 (0.8–6.8)	0.09
∑chlordanes	cases/controls	24/26	33/26	45/24		12/26	19/26	68/24	
	Model 1	1.0	1.1 (0.5–2.5)	1.4 (0.6–3.2)	0.36	1.0	0.8 (0.3–2.4)	2.8 (1.1–7.6)	0.01
	Model 2	1.0	1.0 (0.4–2.4)	1.0 (0.4–2.4)	0.97	1.0	0.7 (0.2–2.1)	2.3 (0.8–6.5)	0.03
	Model 3	1.0	1.0 (0.4–2.4)	1.0 (0.4–2.4)	0.94	1.0	0.7 (0.2–2.2)	2.3 (0.8–6.7)	0.02
∑heptachlor	cases/controls	42/40	5/12	55/24		24/40	6/12	69/24	
	Model 1	1.0	0.6 (0.2–1.8)	2.3 (1.2–4.5)	0.02	1.0	3.1 (0.7–13.2)	6.5 (2.8–14.8)	<0.01
	Model 2	1.0	0.4 (0.1–1.4)	1.9 (0.9–3.8)	0.09	1.0	2.6 (0.5–12.0)	6.2 (2.6–14.7)	<0.01
	Model 3	1.0	0.4 (0.1–1.3)	1.8 (0.9–3.7)	0.11	1.0	2.7 (0.6–13.2)	6.5 (2.7–15.6)	<0.01
PCB subgroups									
∑low-chlorinated PCBs	cases/controls	48/37	9/15	45/24		49/37	9/15	41/24	
	Model 1	1.0	0.7 (0.3–1.8)	2.4 (1.1–5.0)	0.02	1.0	1.7 (0.5–5.3)	4.0 (1.7–9.5)	<0.01
	Model 2	1.0	0.6 (0.2–1.7)	2.5 (1.2–5.3)	0.02	1.0	1.6 (0.5–5.2)	3.7 (1.5–9.1)	<0.01
	Model 3	1.0	0.6 (0.2–1.7)	2.5 (1.1–5.4)	0.02	1.0	1.7 (0.5–5.7)	4.0 (1.6–10.0)	<0.01
∑mid-chlorinated PCBs	cases/controls	21/26	21/26	60/24		17/26	33/26	49/24	
	Model 1	1.0	0.8 (0.3–1.8)	2.0 (0.9–4.6)	0.04	1.0	1.2 (0.4–3.1)	1.4 (0.6–3.7)	0.54
	Model 2	1.0	0.8 (0.3–1.9)	1.8 (0.7–4.1)	0.13	1.0	1.3 (0.5–3.6)	1.3 (0.5–3.5)	0.72
	Model 3	1.0	0.9 (0.4–2.1)	1.8 (0.8–4.4)	0.13	1.0	1.3 (0.5–3.6)	1.3 (0.5–3.5)	0.76
∑high-chlorinated PCBs	cases/controls	18/26	23/26	61/24		18/26	19/26	62/24	
	Model 1	1.0	0.9 (0.4–2.1)	1.9 (0.8–4.6)	0.12	1.0	0.8 (0.3–2.3)	1.5 (0.5–4.2)	0.35
	Model 2	1.0	0.9 (0.4–2.4)	1.9 (0.7–4.9)	0.15	1.0	0.9 (0.3–2.7)	1.4 (0.5–4.1)	0.44
	Model 3	1.0	1.0 (0.4–2.6)	2.1 (0.8–5.6)	0.11	1.0	0.9 (0.3–2.6)	1.4 (0.5–4.2)	0.44

Model 1, adjusted for age and sex; Model 2, further adjusted for family history, body mass index, cigarette smoking, alcohol drinking, and physical activity; Model 3, further adjusted for meat consumption, diabetes and fiber intake. ∑DDTs = rank sum of *o*,*p'*-DDE, *p*,*p'*-DDE, *o*,*p'-*DDT, and *p*,*p'*-DDT; ∑chlordanes = rank sum of *trans*-chlordane, oxychlordane, *trans*-nonachlor, and *cis*-nonachlor; ∑heptachlor = rank sum of heptachlor epoxide and heptachlor; ∑low-chlorinated PCBs (three to four chlorides) = rank sum of PCB18, PCB28, PCB33, and PCB52; ∑mid-chlorinated PCBs (five to six chlorides) = rank sum of PCB101, PCB105, PCB118, PCB138, and PCB153; ∑high-chlorinated PCBs (seven or more chlorides) = rank sum of PCB170, PCB180, PCB187, PCB194, and PCB19. DDE, dichlorodiphenyldichloroethylene; DDT, dichlorodiphenyltrichloroethane; OCP, organochlorine pesticide; PCB, polychlorinated biphenyl; POP, persistent organic pollutant.

The wet-weight concentrations of POPs, OCPs, and PCBs showed similar but slightly weaker associations than those of the lipid-standardized concentrations (S4 and S5 Tables). Due to the large difference in age distribution between the cancer group and the others, the associations were also evaluated only among study subjects in their 50s or 60s. Despite the small number of cases, the similar results were observed (S6 Table).

## Discussion

This study demonstrated that serum concentrations of POPs were associated with an increased risk of polyps and cancer in the large intestine. The results varied depending on the type of POPs. For example, both polyps and cancer were associated with OCPs, whereas only polyps were mostly associated with PCBs, even though low-chlorinated PCBs were associated with the risk of both polyps and cancer. Generally, compounds with low detection rates tended to show clearer results than compounds with high detection rates. Although the assessment of POPs contained in feces would be the most relevant measure of exposure for the determination of the risk of colorectal cancer, strong correlations have been reported for POP concentrations between serum and feces [30, 31]. In support of our findings, the chronic dietary treatment of low dose POP mixtures, similar to human exposure, increased intestinal tumorigenesis in a mouse model [32].

It is important to note that findings on POPs in humans should be interpreted from the viewpoint of mixtures, not individual compounds, because general populations are simultaneously exposed to OCPs and PCBs as a mixture, and serum concentrations of these chemicals are generally positively correlated [33]. In addition, the findings may reflect the effects of even other chemicals, which were not measured in the current study but coexist with OCPs and PCBs. In fact, the serum concentrations of OCPs and PCBs in the current general population should be considered as a surrogate marker of the concentrations of various lipophilic chemicals that are accumulated in adipose tissue and released from adipocytes to circulation through lipolysis [34]. Therefore, the current findings on POPs can be interpreted as the carcinogenic potential of lipophilic chemical mixtures.

In this respect, our findings support the conclusion of the Halifax project [8]. In a literature-based review on 85 common chemicals, which are not currently considered to be human carcinogens, each of these chemicals induced some of the hallmarks of cancer [35, 36], and it was concluded that the cumulative effects of individual chemicals, acting on different pathways, could plausibly result in carcinogenic synergies [8].

In addition, there are reciprocal interactions between POPs in feces and gut microbiota [37]. Therefore, the change of gut microbiota could be one possible mechanism linking POPs and colorectal cancer because the gut microbiota is strongly associated with colorectal carcinogenesis by multiple mechanisms [38]. Furthermore, several in-vitro studies reported that DDT or DDE can induce colorectal adenocarcinoma cell proliferation by oxidative stress or Wnt/β-catenin signaling [39, 40].

One important feature of the carcinogenic potential of low-dose chemical mixtures is non-monotonic dose-response relationships with the risk of carcinogenesis [8]. Under the situation of an inverted U-shaped association, the most common example of non-monotonic dose-response relationships, populations with low-dose exposure can show a linear dose-response relationship because the exposure range would correspond to the linear part of the dose-response relationship [9]. However, populations with high-dose exposure can fail to reveal any association because the exposure range would be in the saturated part of the dose-response [9]. This may explain why the current study subjects with low-dose exposure range showed much stronger associations than those reported in previous case-control studies [19, 20], which examined the exposure to 10 to 100 times higher concentrations of OCPs or PCBs.

The importance of studying general populations with low-dose exposure was supported by the findings that chemicals with low detection rates tended to show stronger associations than those with high detection rates because concentrations of chemicals with low detection rates would be typically lower than those of chemicals with high detection rates. Although prospective studies are required to confirm the current findings, researchers should consider that measurement of these chemicals in established cohorts with high serum concentrations of POPs at baseline would be undesirable.

On the other hand, in the subjects from the current study, the serum concentrations of individual compounds with high detection rates were highly correlated, while those of compounds with low detection rates were not clearly correlated. In fact, the serum concentrations of low-chlorinated PCBs weakly correlated with those of OCPs and other PCBs, suggesting differences in the exposure source and/or toxicodynamics in the subjects from the current study. While fatty animal food has been known as the main external source of exposure to POPs, owing to a higher potential for bioaccumulation and biomagnification along the food chain [41], the exposure source of low-chlorinated PCBs, containing four or fewer chlorine substituents, is airborne [42].

The current study had several limitations. First, the status of the case-control study made it difficult to determine the temporal relationship between POPs and colorectal cancer. However, considering long half-lives of POPs, their serum concentrations can be interpreted as reflecting long-term POP exposure, before the diagnosis of cancer or polyps. Since weight loss before the cancer diagnosis can increase serum concentrations of POPs, we included the weight change during the past year as a possible covariate, but this did not change the results. Second, many ORs of less than 2.0 were statistically non-significant because the current sample size could detect ORs of 2.5–3.0 with an 80% power. Future studies with larger sample sizes would be desirable. Third, subjects with heterogeneous histological types of polyps and heterogenous anatomical sites of cancer were included. It is plausible to assume that the risk might be different, depending on histological types or anatomical sites. Forth, the distribution of age and gender differed significantly among the three study groups. Because it is known that serum POP concentrations vary depending on the age and gender, we had originally planned to assemble study groups with a balanced age and gender distribution. However, at the recruitment stage, such a balance was practically very difficult to achieve; therefore, we changed our plan and adjusted these factors at analyses stage. Fifth, study participants were recruited among persons who visited one university hospital. Therefore, there was a possibility of selection bias, potentially affecting the generalization of our results.

## Conclusion

The current study suggests that the chronic exposure to low-dose POPs is associated with an increased risk of colorectal polyps and cancer. Although this study measured individual OCP and PCB compounds, findings from human studies about POPs should be interpreted from the viewpoint of lipophilic chemical mixtures, which are accumulated in adipose tissue and released from adipocytes to circulation through lipolysis. Prospective studies in general populations with exposure to low-dose POPs are needed to confirm the association between POPs and colorectal cancer.

## Supporting information

S1 Table STROBE statement—Checklist of items that should be included in reports of case-control studies. (DOCX)

S2 Table Distribution of serum concentrations of persistent organic pollutants among study participants (n = 277). (DOCX)

S3 Table Spearman correlation coefficients between summary measures of persistent organic pollutants. (DOCX)

S4 Table Associations between the summary measures of wet-weight concentrations of persistent organic pollutants, organochlorine pesticides, and polychlorinated biphenyls and the risk of colorectal polyps and cancer, calculated using polychotomous logistic regression (Odds ratios and 95% confidence intervals). (DOCX)

S5 Table Associations between the summary measures of wet-weight concentrations of subgroups of organochlorine pesticides and polychlorinated biphenyls and the risk of colorectal polyps or cancer, calculated using polychotomous logistic regression (Odds ratios and 95% confidence intervals). (DOCX)

S6 Table Associations between the summary measures of persistent organic pollutants, organochlorine pesticides, and polychlorinated biphenyls and the risk of colorectal polyps or cancer, calculated using polychotomous logistic regression according to the age group (Odds ratios and 95% confidence intervals). (DOCX)

S1 Fig How to calculate summary measure of POPs. (PDF)

S1 File Data set. (XLS)
