# Supplementary material for: Association of colorectal polyps and cancer with low-dose persistent organic pollutants: A case-control study
Source: PLoS One. 2018 Dec 6;13(12):e0208546. doi: 10.1371/journal.pone.0208546 (PMC6283632; doi:10.1371/journal.pone.0208546)
Supplement: S2 Table — (DOCX) [file pone.0208546.s002.docx]

**S2 Table**

Distribution of serum concentrations of persistent organic pollutants among study participants (n = 277).

| Individual POPs | Detection rate (%) | Lipid-standardized concentration (ng/g of lipid) | | | | | | |
| --- | --- | --- | --- | --- | --- | --- | --- | --- |
|  |  | Minimum | 10 percentile | 25 percentile | 50 percentile | 75 percentile | 90 percentile | Maximum |
| *α*-HCH | 1.1 | 166.67 | 166.67 | 166.67 | 166.67 | 166.67 | 166.67 | 3496.09 |
| *β*-HCH | 96.8 | 166.67 | 7234.64 | 11025.99 | 18064.02 | 28620.37 | 47634.58 | 123895.67 |
| *γ*-HCH | 3.3 | 166.67 | 166.67 | 166.67 | 166.67 | 166.67 | 166.67 | 4181.80 |
| *δ*-HCH | 0.0 | NA | NA | NA | NA | NA | NA | NA |
| *o,p′*-DDE | 13.4 | 166.67 | 166.67 | 166.67 | 166.67 | 166.67 | 2319.35 | 11538.09 |
| *p,p′*-DDE | 99.6 | 166.67 | 82344.34 | 114400.75 | 164974.26 | 299253.82 | 461554.30 | 1475702.39 |
| *o,p′*-DDD | 0.4 | 166.67 | 166.67 | 166.67 | 166.67 | 166.67 | 166.67 | 2082.03 |
| *p,p′*-DDD | 7.2 | 166.67 | 166.67 | 166.67 | 166.67 | 166.67 | 166.67 | 7808.96 |
| *o,p′*-DDT | 13.7 | 166.67 | 166.67 | 166.67 | 166.67 | 166.67 | 1986.46 | 5726.33 |
| *p,p′*-DDT | 91.3 | 166.67 | 3021.00 | 6204.20 | 9678.14 | 14290.96 | 19094.51 | 60861.47 |
| *trans*-chlordane | 12.6 | 66.67 | 66.67 | 66.67 | 66.67 | 66.67 | 925.41 | 5051.46 |
| *cis*-chlordane | 1.8 | 66.67 | 66.67 | 66.67 | 66.67 | 66.67 | 66.67 | 1423.04 |
| oxychlordane | 70.4 | 66.67 | 66.67 | 66.67 | 2900.79 | 5036.05 | 7963.21 | 21403.22 |
| *trans*-nonachlor | 96.4 | 66.67 | 3163.27 | 5064.81 | 7696.74 | 12087.22 | 17557.19 | 59874.11 |
| *cis*-nonachlor | 58.8 | 66.67 | 66.67 | 66.67 | 1138.13 | 2253.19 | 3642.93 | 11722.20 |
| heptachlor epoxide | 57.4 | 166.67 | 166.67 | 166.67 | 2002.23 | 4060.28 | 6499.57 | 29843.81 |
| heptachlor | 17.0 | 166.67 | 166.67 | 166.67 | 166.67 | 166.67 | 2485.33 | 187417.03 |
| PCB18 | 36.1 | 83.33 | 83.33 | 83.33 | 83.33 | 442.41 | 1138.93 | 3728.84 |
| PCB28 | 21.7 | 83.33 | 83.33 | 83.33 | 83.33 | 83.33 | 2859.60 | 21230.66 |
| PCB33 | 12.6 | 83.33 | 83.33 | 83.33 | 83.33 | 83.33 | 144.84 | 6933.67 |
| PCB44 | 7.6 | 83.33 | 83.33 | 83.33 | 83.33 | 83.33 | 83.33 | 15728.41 |
| PCB52 | 30.0 | 83.33 | 83.33 | 83.33 | 83.33 | 345.50 | 1496.96 | 8881.84 |
| PCB70 | 3.3 | 83.33 | 83.33 | 83.33 | 83.33 | 83.33 | 83.33 | 16972.85 |
| PCB101 | 24.6 | 83.33 | 83.33 | 83.33 | 83.33 | 83.33 | 489.15 | 11001.53 |
| PCB105 | 68.6 | 83.33 | 83.33 | 83.33 | 873.38 | 1551.67 | 2163.84 | 5531.16 |
| PCB118 | 95.7 | 83.33 | 1199.21 | 2414.80 | 4275.14 | 6473.25 | 9663.50 | 28959.13 |
| PCB128 | 8.3 | 83.33 | 83.33 | 83.33 | 83.33 | 83.33 | 83.33 | 9811.07 |
| PCB138 | 92.4 | 166.67 | 4339.63 | 7628.86 | 11990.31 | 20690.15 | 29317.78 | 95133.99 |
| PCB153 | 100.0 | 3845.41 | 10130.65 | 13932.32 | 21071.22 | 33536.97 | 46670.48 | 180239.62 |
| PCB170 | 89.9 | 166.67 | 166.67 | 2676.50 | 4319.51 | 7177.65 | 10768.87 | 26222.83 |
| PCB180 | 99.6 | 166.67 | 4542.07 | 7771.54 | 13568.98 | 23512.10 | 33691.93 | 102226.17 |
| PCB187 | 95.3 | 166.67 | 2299.50 | 4045.94 | 6026.86 | 10144.47 | 13987.78 | 48786.66 |
| PCB194 | 55.6 | 166.67 | 166.67 | 166.67 | 1283.99 | 3171.10 | 5755.25 | 27951.58 |
| PCB195 | 0.0 | NA | NA | NA | NA | NA | NA | NA |
| PCB199 | 71.1 | 166.67 | 166.67 | 166.67 | 2033.21 | 3484.11 | 5345.29 | 14274.14 |
| PCB206 | 1.8 | 727.56 | 833.33 | 833.33 | 833.33 | 833.33 | 833.33 | 2240.32 |

POP concentrations below LOD were replaced with LOD/3 values.

DDD, dichlorodiphenyldichloroethane; DDE, dichlorodiphenyldichloroethylene; DDT, dichlorodiphenyltrichloroethane; HCH, hexachlorocyclohexane; LOD, limit of detection; NA, not applicable; PCB, polychlorinated biphenyl; POP, persistent organic pollutant.
