# Supplementary material for: Association of colorectal polyps and cancer with low-dose persistent organic pollutants: A case-control study
Source: PLoS One. 2018 Dec 6;13(12):e0208546. doi: 10.1371/journal.pone.0208546 (PMC6283632; doi:10.1371/journal.pone.0208546)
Supplement: S3 Table — (DOCX) [file pone.0208546.s003.docx]

**S3 Table**

Spearman correlation coefficients between summary measures of persistent organic pollutants.

|  | ∑POPs | ∑OCPs | ∑DDTs | ∑chlordanes | ∑heptachlor | ∑PCBs | ∑low-  chlorinated  PCBs | ∑mid-  chlorinated  PCBs | ∑high-  chlorinated  PCBs |
| --- | --- | --- | --- | --- | --- | --- | --- | --- | --- |
| ∑POPs | 1.00 | 0.88 | 0.77 | 0.79 | 0.58 | 0.94 | 0.32 | 0.89 | 0.80 |
|  |  | (<0.01) | (<0.01) | (<0.01) | (<0.01) | (<0.01) | (<0.01) | (<0.01) | (<0.01) |
| ∑OCPs |  | 1.00 | 0.84 | 0.90 | 0.71 | 0.67 | 0.13 | 0.73 | 0.60 |
|  |  |  | (<0.01) | (<0.01) | (<0.01) | (<0.01) | (0.03) | (<0.01) | (<0.01) |
| ∑DDTs |  |  | 1.00 | 0.64 | 0.42 | 0.62 | 0.10 | 0.68 | 0.55 |
|  |  |  |  | (<0.01) | (<0.01) | (<0.01) | (0.11) | (<0.01) | (<0.01) |
| ∑chlordanes |  |  |  | 1.00 | 0.55 | 0.61 | 0.11 | 0.64 | 0.57 |
|  |  |  |  |  | (<0.01) | (<0.01) | (0.07) | (<0.01) | (<0.01) |
| ∑heptachlor |  |  |  |  | 1.00 | 0.43 | 0.21 | 0.43 | 0.32 |
|  |  |  |  |  |  | (<0.01) | (<0.01) | (<0.01) | (<0.01) |
| ∑PCBs |  |  |  |  |  | 1.00 | 0.41 | 0.87 | 0.84 |
|  |  |  |  |  |  |  | (<0.01) | (<0.01) | (<0.01) |
| ∑low-chlorinated PCBs |  |  |  |  |  |  | 1.00 | 0.15 | -0.01 |
|  |  |  |  |  |  |  |  | (0.01) | (0.85) |
| ∑mid-chlorinated PCBs |  |  |  |  |  |  |  | 1.00 | 0.71 |
|  |  |  |  |  |  |  |  |  | (<0.01) |
| ∑high-chlorinated PCBs |  |  |  |  |  |  |  |  | 1.00 |
|  |  |  |  |  |  |  |  |  |  |

∑POPs = ∑OCPs + ∑PCBs; ∑OCPs = β-hexachlorocyclohexane + ∑DDTs + ∑chlordanes + ∑heptachlor; ∑DDTs = rank sum of *o,p'*-DDE, *p,p'*-DDE, *o,p'-*DDT, and *p,p'*-DDT; ∑chlordanes = rank sum of *trans*-chlordane, oxychlordane, *trans*-nonachlor, and *cis*-nonachlor; ∑heptachlor = rank sum of heptachlor epoxide and heptachlor; ∑PCBs = ∑low-chlorinated PCBs + ∑mid-chlorinated PCBs + ∑high-chlorinated PCBs; ∑low-chlorinated PCBs (three to four chlorides) = rank sum of PCB18, PCB28, PCB33, and PCB52; ∑mid-chlorinated PCBs (five to six chlorides) = rank sum of PCB101, PCB105, PCB118, PCB138, and PCB153; ∑high-chlorinated PCBs (seven or more chlorides) = rank sum of PCB170, PCB180, PCB187, PCB194, and PCB19.

DDE, dichlorodiphenyldichloroethylene; DDT, dichlorodiphenyltrichloroethane; OCP, organochlorine pesticide; PCB, polychlorinated biphenyl; POP, persistent organic pollutant.
