# Supplementary material for: Association of colorectal polyps and cancer with low-dose persistent organic pollutants: A case-control study
Source: PLoS One. 2018 Dec 6;13(12):e0208546. doi: 10.1371/journal.pone.0208546 (PMC6283632; doi:10.1371/journal.pone.0208546)
Supplement: S4 Table — (DOCX) [file pone.0208546.s004.docx]

**S4 Table**

Associations between the summary measures of wet-weight concentrations of persistent organic pollutants, organochlorine pesticides, and polychlorinated biphenyls and the risk of colorectal polyps and cancer, calculated using polychotomous logistic regression (Odds ratios and 95% confidence intervals).

| Measure | Model | Dependent variables: colorectal polyps | | | *P*_trend_ | | Dependent variables: colorectal cancer | | | *P*_trend_ |
| --- | --- | --- | --- | --- | --- | --- | --- | --- | --- | --- |
|  |  | 1^st^ tertile | 2^nd^ tertile | 3^rd^ tertile |  | | 1^st^ tertile | 2^nd^ tertile | 3^rd^ tertile |  |
| ∑ POPs | cases/controls | 18/26 | 20/26 | 64/24 | |  | 18/26 | 32/26 | 49/24 |  |
|  | Model 1 | 1.0 | 0.7 (0.3-1.8) | 2.5 (1.1-5.7) | | 0.01 | 1.0 | 0.7 (0.3-2.0) | 1.5 (0.6-4.0) | 0.29 |
|  | Model 2 | 1.0 | 0.7 (0.3-1.9) | 2.3 (1.0-5.3) | | 0.03 | 1.0 | 0.7 (0.2-1.9) | 1.4 (0.5-3.7) | 0.44 |
|  | Model 3 | 1.0 | 0.8 (0.3-2.0) | 2.4 (1.0-5.7) | | 0.02 | 1.0 | 0.7 (0.2-2.0) | 1.4 (0.5-3.9) | 0.41 |
|  | Model 4 | 1.0 | 0.8 (0.3-2.1) | 2.1 (0.9-5.2) | | 0.06 | 1.0 | 0.8 (0.3-2.5) | 1.6 (0.6-4.8) | 0.30 |
| ∑ OCPs | cases/controls | 20/26 | 22/26 | 60/24 | |  | 12/26 | 23/26 | 64/24 |  |
|  | Model 1 | 1.0 | 0.9 (0.4-2.2) | 2.6 (1.2-5.9) | | 0.01 | 1.0 | 0.6 (0.2-2.0) | 2.7 (1.0-7.2) | 0.01 |
|  | Model 2 | 1.0 | 0.8 (0.3-2.1) | 2.2 (0.9-5.4) | | 0.04 | 1.0 | 0.7 (0.2-2.2) | 2.5 (0.9-7.3) | 0.02 |
|  | Model 3 | 1.0 | 0.8 (0.3-2.1) | 2.2 (0.9-5.5) | | 0.04 | 1.0 | 0.7 (0.2-2.3) | 2.7 (0.9-7.8) | 0.02 |
|  | Model 4 | 1.0 | 0.8 (0.3-2.0) | 2.0 (0.8-4.9) | | 0.11 | 1.0 | 0.7 (0.2-2.4) | 3.0 (1.0-9.5) | 0.01 |
| ∑ PCBs | cases/controls | 16/26 | 18/26 | 68/24 | |  | 26/26 | 26/26 | 47/24 |  |
|  | Model 1 | 1.0 | 0.8 (0.3-2.0) | 2.9 (1.3-6.8) | | <0.01 | 1.0 | 0.7 (0.3-1.8) | 1.0 (0.4-2.6) | 0.96 |
|  | Model 2 | 1.0 | 0.8 (0.3-2.2) | 2.5 (1.1-6.2) | | 0.02 | 1.0 | 0.7 (0.3-2.0) | 0.9 (0.3-2.4) | 0.85 |
|  | Model 3 | 1.0 | 0.9 (0.3-2.2) | 2.6 (1.1-6.5) | | 0.02 | 1.0 | 0.7 (0.3-2.0) | 0.9 (0.3-2.5) | 0.88 |
|  | Model 4 | 1.0 | 0.8 (0.3-2.2) | 2.4 (1.0-5.9) | | 0.03 | 1.0 | 0.8 (0.3-2.5) | 1.1 (0.4-3.0) | 0.92 |

Model 1, adjusted for age and sex; Model 2: further adjusted for family history, body mass index, cigarette smoking, alcohol drinking, and physical activity; Model 3: further adjusted for meat consumption, diabetes and fiber intake; Model 4: further adjusted for total cholesterol and triglyceride.

∑POPs = ∑OCPs + ∑PCBs; ∑OCPs = β-hexachlorocyclohexane + ∑DDTs + ∑chlordanes + ∑heptachlor; ∑PCBs = ∑low-chlorinated PCBs + ∑mid-chlorinated PCBs + ∑high-chlorinated PCBs.

DDT, dichlorodiphenyltrichloroethane; OCP, organochlorine pesticide; PCB, polychlorinated biphenyl; POP, persistent organic pollutant.
