# Supplementary material for: Association of colorectal polyps and cancer with low-dose persistent organic pollutants: A case-control study
Source: PLoS One. 2018 Dec 6;13(12):e0208546. doi: 10.1371/journal.pone.0208546 (PMC6283632; doi:10.1371/journal.pone.0208546)
Supplement: S5 Table — (DOCX) [file pone.0208546.s005.docx]

**S5 Table**

Associations between the summary measures of wet-weight concentrations of subgroups of organochlorine pesticides and polychlorinated biphenyls and the risk of colorectal polyps or cancer, calculated using polychotomous logistic regression (Odds ratios and 95% confidence intervals).

| Measures | Model | Dependent variables: colorectal polyps | | | *P*_trend_ | Dependent variables: colorectal cancer | | | *P*_trend_ |
| --- | --- | --- | --- | --- | --- | --- | --- | --- | --- |
|  |  | 1^st^ tertile | 2^nd^ tertile | 3^rd^ tertile |  | 1^st^ tertile | 2^nd^ tertile | 3^rd^ tertile |  |
| OCP subgroups |  |  |  |  |  |  |  |  |  |
| β-hexachlorocyclohexane | cases/controls | 18/26 | 22/26 | 62/24 |  | 13/26 | 35/26 | 51/24 |  |
|  | Model 1 | 1.0 | 1.5 (0.6-3.5) | 4.9 (2.0-12.1) | <0.01 | 1.0 | 2.7 (1.0-7.7) | 1.4 (0.5-3.9) | 0.99 |
|  | Model 2 | 1.0 | 1.4 (0.5-3.6) | 5.3 (2.0-14.4) | <0.01 | 1.0 | 3.7 (1.2-11.5) | 1.9 (0.6-5.9) | 0.69 |
|  | Model 3 | 1.0 | 1.5 (0.6-3.9) | 5.6 (2.0-15.6) | <0.01 | 1.0 | 3.8 (1.2-11.8) | 1.9 (0.6-6.0) | 0.69 |
|  | Model 4 | 1.0 | 1.4 (0.5-3.8) | 5.2 (1.8-15.0) | <0.01 | 1.0 | 3.5 (1.1-11.6) | 2.0 (0.6-6.9) | 0.54 |
| ∑ DDTs | cases/controls | 27/26 | 19/26 | 56/24 |  | 14/26 | 21/26 | 54/24 |  |
|  | Model 1 | 1.0 | 0.6 (0.2-1.3) | 1.7 (0.8-3.6) | 0.11 | 1.0 | 0.6 (0.2-1.7) | 1.7 (0.7-4.0) | 0.18 |
|  | Model 2 | 1.0 | 0.6 (0.2-1.4) | 1.4 (0.6-3.2) | 0.30 | 1.0 | 0.5 (0.2-1.4) | 1.4 (0.6-3.5) | 0.34 |
|  | Model 3 | 1.0 | 0.6 (0.3-1.5) | 1.5 (0.7-3.5) | 0.25 | 1.0 | 0.5 (0.2-1.5) | 1.4 (0.5-3.5) | 0.35 |
|  | Model 4 | 1.0 | 0.6 (0.2-1.5) | 1.3 (0.5-3.1) | 0.47 | 1.0 | 0.6 (0.2-1.8) | 1.6 (0.6-4.5) | 0.23 |
| ∑ chlordanes | cases/controls | 22/26 | 31/26 | 49/24 |  | 17/26 | 16/26 | 66/24 |  |
|  | Model 1 | 1.0 | 1.1 (0.5-2.6) | 1.8 (0.8-3.9) | 0.14 | 1.0 | 0.5 (0.2-1.3) | 2.3 (0.9-6.0) | 0.01 |
|  | Model 2 | 1.0 | 1.0 (0.4-2.4) | 1.3 (0.5-3.0) | 0.61 | 1.0 | 0.4 (0.1-1.4) | 2.2 (0.8-6.0) | 0.02 |
|  | Model 3 | 1.0 | 1.0 (0.4-2.3) | 1.2 (0.5-2.9) | 0.69 | 1.0 | 0.5 (0.1-1.4) | 2.2 (0.8-6.3) | 0.02 |
|  | Model 4 | 1.0 | 1.0 (0.4-2.4) | 1.0 (0.4-2.4) | 0.90 | 1.0 | 0.5 (0.1-1.6) | 2.6 (0.8-7.8) | 0.02 |
| ∑ heptachlor | cases/controls | 40/40 | 3/12 | 59/24 |  | 26/40 | 5/12 | 68/24 |  |
|  | Model 1 | 1.0 | 0.3 (0.1-1.3) | 2.8 (1.4-5.5) | <0.01 | 1.0 | 2.2 (0.5-9.7) | 7.2 (3.1-16.7) | <0.01 |
|  | Model 2 | 1.0 | 0.2 (0.1-1.0) | 2.4 (1.2-5.0) | 0.02 | 1.0 | 1.9 (0.4-8.9) | 7.3 (3.0-17.7) | <0.01 |
|  | Model 3 | 1.0 | 0.2 (0.0-1.0) | 2.3 (1.1-4.8) | 0.03 | 1.0 | 2.0 (0.4-9.8) | 7.6 (3.1-18.9) | <0.01 |
|  | Model 4 | 1.0 | 0.2 (0.0-1.0) | 2.1 (1.0-4.4) | 0.06 | 1.0 | 1.7 (0.3-9.0) | 8.2 (3.2-21.2) | <0.01 |
| PCB subgroups |  |  |  |  |  |  |  |  |  |
| ∑ low-chlorinated PCBs | cases/controls | 49/37 | 9/17 | 44/22 |  | 50/37 | 22/17 | 27/22 |  |
|  | Model 1 | 1.0 | 0.6 (0.2-1.6) | 2.4 (1.1-5.0) | 0.02 | 1.0 | 2.9 (1.1-8.0) | 3.2 (1.3-8.1) | <0.01 |
|  | Model 2 | 1.0 | 0.6 (0.2-1.5) | 2.4 (1.1-5.2) | 0.03 | 1.0 | 2.6 (0.9-7.3) | 3.2 (1.2-8.0) | 0.02 |
|  | Model 3 | 1.0 | 0.6 (0.2-1.6) | 2.4 (1.1-5.2) | 0.03 | 1.0 | 2.8 (1.0-7.9) | 3.4 (1.3-8.8) | <0.01 |
|  | Model 4 | 1.0 | 0.5 (0.2-1.5) | 2.2 (1.0-4.9) | 0.05 | 1.0 | 2.5 (0.9-7.5) | 3.2 (1.2-8.3) | 0.02 |
| ∑ mid-chlorinated PCBs | cases/controls | 21/26 | 27/26 | 54/24 |  | 27/26 | 37/26 | 35/24 |  |
|  | Model 1 | 1.0 | 1.0 (0.4-2.3) | 1.9 (0.8-4.2) | 0.09 | 1.0 | 1.0 (0.4-2.4) | 0.7 (0.3-1.9) | 0.44 |
|  | Model 2 | 1.0 | 1.0 (0.4-2.3) | 1.5 (0.6-3.5) | 0.30 | 1.0 | 0.9 (0.3-2.3) | 0.6 (0.2-1.6) | 0.25 |
|  | Model 3 | 1.0 | 1.0 (0.4-2.3) | 1.5 (0.6-3.5) | 0.33 | 1.0 | 0.9 (0.3-2.4) | 0.6 (0.2-1.6) | 0.23 |
|  | Model 4 | 1.0 | 0.9 (0.4-2.2) | 1.2 (0.5-3.0) | 0.59 | 1.0 | 1.1 (0.4-3.0) | 0.7 (0.2-1.9) | 0.36 |
| ∑ high-chlorinated PCBs | cases/controls | 16/26 | 20/26 | 66/24 |  | 23/26 | 19/26 | 57/24 |  |
|  | Model 1 | 1.0 | 0.9 (0.4-2.3) | 2.6 (1.1-6.3) | 0.02 | 1.0 | 0.6 (0.2-1.7) | 1.2 (0.4-3.2) | 0.63 |
|  | Model 2 | 1.0 | 1.0 (0.4-2.6) | 2.4 (0.9-6.2) | 0.05 | 1.0 | 0.7 (0.2-1.9) | 1.1 (0.4-3.1) | 0.74 |
|  | Model 3 | 1.0 | 1.1 (0.4-2.8) | 2.6 (1.0-6.8) | 0.04 | 1.0 | 0.6 (0.2-1.9) | 1.1 (0.4-3.2) | 0.71 |
|  | Model 4 | 1.0 | 1.1 (0.4-2.8) | 2.3 (0.9-6.2) | 0.08 | 1.0 | 0.8 (0.3-2.4) | 1.4 (0.5-4.4) | 0.46 |

Model 1, adjusted for age and sex; Model 2: further adjusted for family history, body mass index, cigarette smoking, alcohol drinking, and physical activity; Model 3: further adjusted for meat consumption, diabetes and fiber intake; Model 4: further adjusted for total cholesterol and triglyceride.

∑DDTs = rank sum of *o,p'*-DDE, *p,p'*-DDE, *o,p'-*DDT, and *p,p'*-DDT; ∑chlordanes = rank sum of *trans*-chlordane, oxychlordane, *trans*-nonachlor, and *cis*-nonachlor; ∑heptachlor = rank sum of heptachlor epoxide and heptachlor; ∑low-chlorinated PCBs (three to four chlorides) = rank sum of PCB18, PCB28, PCB33, and PCB52; ∑mid-chlorinated PCBs (five to six chlorides) = rank sum of PCB101, PCB105, PCB118, PCB138, and PCB153; ∑high-chlorinated PCBs (seven or more chlorides) = rank sum of PCB170, PCB180, PCB187, PCB194, and PCB19.

DDE, dichlorodiphenyldichloroethylene; DDT, dichlorodiphenyltrichloroethane; OCP, organochlorine pesticide; PCB, polychlorinated biphenyl; POP, persistent organic pollutant.
