# Supplementary material for: Association of colorectal polyps and cancer with low-dose persistent organic pollutants: A case-control study
Source: PLoS One. 2018 Dec 6;13(12):e0208546. doi: 10.1371/journal.pone.0208546 (PMC6283632; doi:10.1371/journal.pone.0208546)
Supplement: S6 Table — (DOCX) [file pone.0208546.s006.docx]

**S6 Table**

Associations between the summary measures of persistent organic pollutants, organochlorine pesticides, and polychlorinated biphenyls and the risk of colorectal polyps or cancer, calculated using polychotomous logistic regression according to the age group (Odds ratios and 95% confidence intervals).

| Measures | Model | Dependent variables: colorectal polyps | | | *P*_trend_ | Dependent variables: colorectal cancer | | | *P*_trend_ |
| --- | --- | --- | --- | --- | --- | --- | --- | --- | --- |
|  |  | 1^st^ tertile | 2^nd^ tertile | 3^rd^ tertile |  | 1^st^ tertile | 2^nd^ tertile | 3^rd^ tertile |  |
| 50 ≤ age <59 |  |  |  |  |  |  |  |  |  |
| ∑POPs | cases/controls | 3/10 | 2/10 | 28/9 |  | 1/10 | 8/10 | 9/9 |  |
|  | Model 1 | 1.0 | 0.7 (0.1-5.4) | 9.8 (2.1-46.1) | <0.01 | 1.0 | 8.3 (0.8-81.6) | 9.5 (1.0-93.1) | 0.07 |
|  | Model 2 | 1.0 | 0.9 (0.1-8.2) | 8.4 (1.5-47.3) | <0.01 | 1.0 | 9.5 (0.8-115.2) | 5.7 (0.5-66.9) | 0.30 |
|  | Model 3 | 1.0 | 0.9 (0.1-9.6) | 11.1 (1.5-84.6) | <0.01 | 1.0 | 6.5 (0.6-77.2) | 3.6 (0.3-44.3) | 0.56 |
| ∑OCPs | cases/controls | 3/11 | 7/9 | 23/9 |  | 3/11 | 6/9 | 9/9 |  |
|  | Model 1 | 1.0 | 4.0 (0.7-22.6) | 13.7 (2.7-70.3) | <0.01 | 1.0 | 3.2 (0.6-17.9) | 4.9 (0.9-26.2) | 0.06 |
|  | Model 2 | 1.0 | 2.5 (0.4-15.8) | 8.4 (1.5-48.0) | 0.01 | 1.0 | 1.9 (0.3-12.6) | 2.4 (0.4-15.3) | 0.37 |
|  | Model 3 | 1.0 | 2.1 (0.3-15.9) | 9.6 (1.3-68.4) | 0.01 | 1.0 | 1.8 (0.2-14.2) | 2.2 (0.3-15.3) | 0.46 |
| ∑PCBs | cases/controls | 2/10 | 4/10 | 27/9 |  | 2/10 | 10/10 | 6/9 |  |
|  | Model 1 | 1.0 | 1.7 (0.2-11.9) | 11.2 (2.0-63.5) | <0.01 | 1.0 | 4.3 (0.7-25.7) | 2.6 (0.4-16.9) | 0.48 |
|  | Model 2 | 1.0 | 1.4 (0.2-12.6) | 12.5 (1.9-82.8) | <0.01 | 1.0 | 5.5 (0.8-39.6) | 1.4 (0.2-12.9) | 0.89 |
|  | Model 3 | 1.0 | 2.4 (0.2-31.1) | 23.9 (2.0-282.1) | <0.01 | 1.0 | 3.6 (0.5-25.6) | 0.6 (0.1-6.4) | 0.61 |
| 60 ≤ age <69 |  |  |  |  |  |  |  |  |  |
| ∑POPs | cases/controls | 7/5 | 10/5 | 17/3 |  | 2/5 | 5/5 | 18/3 |  |
|  | Model 1 | 1.0 | 1.0 (0.2-5.2) | 3.0 (0.5-17.3) | 0.19 | 1.0 | 1.9 (0.2-15.9) | 12.1 (1.5-97.1) | 0.01 |
|  | Model 2 | 1.0 | 1.7 (0.2-13.5) | 3.3 (0.5-23.6) | 0.22 | 1.0 | 4.7 (0.4-54.9) | 23.3 (2.2-252.8) | 0.01 |
|  | Model 3 | 1.0 | 1.5 (0.2-12.4) | 3.2 (0.4-22.4) | 0.24 | 1.0 | 4.1 (0.3-52.4) | 21.1 (1.9-237.0) | 0.01 |
| ∑OCPs | cases/controls | 6/5 | 15/5 | 13/3 |  | 4/5 | 2/5 | 19/3 |  |
|  | Model 1 | 1.0 | 2.3 (0.5-11.7) | 4.2 (0.7-25.3) | 0.14 | 1.0 | 0.5 (0.1-4.0) | 9.4 (1.4-60.8) | 0.01 |
|  | Model 2 | 1.0 | 2.5 (0.4-16.6) | 3.4 (0.5-25.5) | 0.29 | 1.0 | 0.4 (0.0-4.8) | 12.3 (1.3-116.6) | 0.01 |
|  | Model 3 | 1.0 | 2.4 (0.4-16.6) | 3.5 (0.5-27.4) | 0.29 | 1.0 | 0.4 (0.0-4.5) | 11.2 (1.1-108.9) | 0.01 |
| ∑PCBs | cases/controls | 8/5 | 6/5 | 20/3 |  | 3/5 | 3/5 | 19/3 |  |
|  | Model 1 | 1.0 | 0.6 (0.1-3.3) | 3.0 (0.5-17.0) | 0.19 | 1.0 | 0.9 (0.1-6.9) | 8.7 (1.3-59.9) | 0.02 |
|  | Model 2 | 1.0 | 1.0 (0.1-7.8) | 3.2 (0.5-21.7) | 0.22 | 1.0 | 1.9 (0.2-19.3) | 13.9 (1.6-121.8) | 0.01 |
|  | Model 3 | 1.0 | 0.8 (0.1-6.8) | 3.4 (0.5-25.9) | 0.22 | 1.0 | 1.3 (0.1-15.0) | 14.7 (1.5-143.3) | 0.01 |

Model 1, adjusted for sex; Model 2, further adjusted for family history, body mass index, cigarette smoking, alcohol drinking, and physical activity; Model 3, further adjusted for meat consumption, diabetes and fiber intake.

∑POPs = ∑OCPs + ∑PCBs; ∑OCPs = β-hexachlorocyclohexane + ∑DDTs + ∑chlordanes + ∑heptachlor; ∑PCBs = ∑low-chlorinated PCBs + ∑mid-chlorinated PCBs + ∑high-chlorinated PCBs.

DDT, dichlorodiphenyltrichloroethane; OCP, organochlorine pesticide; PCB, polychlorinated biphenyl; POP, persistent organic pollutant.
