# Supplementary material for: Association of colorectal polyps and cancer with low-dose persistent organic pollutants: A case-control study
Source: PLoS One. 2018 Dec 6;13(12):e0208546. doi: 10.1371/journal.pone.0208546 (PMC6283632; doi:10.1371/journal.pone.0208546)
Supplement: S1 Fig — (PDF) [file pone.0208546.s007.pdf]

# S1 Fig. How to calculate summary measure of POPs

| Subjects | Concentrations | | |
|---|---|---|---|
| | **Individual POP 1** | **Individual POP 2** | **Individual POP 3** |
| ID001 | 14.2 | 59.0 | 1072.1 |
| ID002 | <LOD | <LOD | 723.4 |
| ID003 | <LOD | 50.6 | 2023.8 |
| ID004 | <LOD | <LOD | 349.4 |
| ID005 | <LOD | 53.4 | 1232.6 |
| ID006 | 13.2 | 128.1 | 1675.3 |
| ID007 | 39.2 | 250.1 | 6267.2 |
| ID008 | <LOD | 125.6 | 808.4 |
| ID009 | 16.6 | 191.3 | 2154.2 |
| ID010 | 18.5 | 156.5 | 1054.7 |

LOD, limit of detection

The concentrations of POPs that were below LOD were substituted with a constant value equal to one-third of LOD.
e.g., LOD of POP1=9, LOD of POP2=15

| Subjects | Concentrations | | |
|---|---|---|---|
| | **Individual POP 1** | **Individual POP 2** | **Individual POP 3** |
| ID001 | 14.2 | 59.0 | 1072.1 |
| ID002 | 3.0 | 5.0 | 723.4 |
| ID003 | 3.0 | 50.6 | 2023.8 |
| ID004 | 3.0 | 5.0 | 349.4 |
| ID005 | 3.0 | 53.4 | 1232.6 |
| ID006 | 13.2 | 128.1 | 1675.3 |
| ID007 | 39.2 | 250.1 | 6267.2 |
| ID008 | 3.0 | 125.6 | 808.4 |
| ID009 | 16.6 | 191.3 | 2154.2 |
| ID010 | 18.5 | 156.5 | 1054.7 |

Sum the concentrations of POPs

| Concentration-based summary measure of POPs | |
|---|---|
| ID001 | 1145.3 |
| ID002 | 731.4 |
| ID003 | 2077.4 |
| ID004 | 357.4 |
| ID005 | 1289 |
| ID006 | 1816.6 |
| ID007 | 6556.5 |
| ID008 | 937 |
| ID009 | 2362.1 |
| ID010 | 1229.7 |

Rank according to the concentrations of individual POPs.

| Subjects | Ranks | | |
|---|---|---|---|
| | **Individual POP 1** | **Individual POP 2** | **Individual POP 3** |
| ID001 | 7 | 5 | 5 |
| ID002 | 3 | 1.5 | 2 |
| ID003 | 3 | 3 | 8 |
| ID004 | 3 | 1.5 | 1 |
| ID005 | 3 | 4 | 6 |
| ID006 | 6 | 7 | 7 |
| ID007 | 10 | 10 | 10 |
| ID008 | 3 | 6 | 3 |
| ID009 | 8 | 9 | 9 |
| ID010 | 9 | 8 | 4 |

In case of The concentrations of POPs that were below LOD, rank of individual POP was substituted with 1.

| Subjects | Ranks | | |
|---|---|---|---|
| | **Individual POP 1** | **Individual POP 2** | **Individual POP 3** |
| ID001 | 7 | 5 | 5 |
| ID002 | 1 | 1 | 2 |
| ID003 | 1 | 3 | 8 |
| ID004 | 1 | 1 | 1 |
| ID005 | 1 | 4 | 6 |
| ID006 | 6 | 7 | 7 |
| ID007 | 10 | 10 | 10 |
| ID008 | 1 | 6 | 3 |
| ID009 | 8 | 9 | 9 |
| ID010 | 9 | 8 | 4 |

Sum the ranks of POPs

| Rank-based summary measure of POPs | |
|---|---|
| ID001 | 17 |
| ID002 | 4 |
| ID003 | 12 |
| ID004 | 3 |
| ID005 | 11 |
| ID006 | 20 |
| ID007 | 30 |
| ID008 | 10 |
| ID009 | 26 |
| ID010 | 21 |
